# Material Characterizations of Gr-Based Magnetorheological Elastomer for Possible Sensor Applications: Rheological and Resistivity Properties

**DOI:** 10.3390/ma12030391

**Published:** 2019-01-27

**Authors:** Muhammad Kashfi Shabdin, Mohd Azizi Abdul Rahman, Saiful Amri Mazlan, Ubaidillah, Norhiwani Mohd Hapipi, Dimas Adiputra, Siti Aishah Abdul Aziz, Irfan Bahiuddin, Seung-Bok Choi

**Affiliations:** 1 Advanced Vehicle System Research Laboratory, Malaysia Japan International Institute of Technology, Universiti Teknologi Malaysia, Jalan Sultan Yahya Petra, Kuala Lumpur 54100, Malaysia; dekashaf@gmail.com (M.K.S.); azizi.kl@utm.my (M.A.A.R.); hiwani87@gmail.com (N.M.H.); adimas2@live.utm.my (D.A.); aishah118@gmail.com (S.A.A.A.); Irfan.bahiuddin@ugm.ac.id (I.B.); 2 Mechanical Engineering, Faculty of Engineering, Universitas Sebelas Maret, Surakarta, Central Java 57126, Indonesia; 3 National Center for Sustainable Transportation Technology (NCSTT), Bandung 40132, Indonesia; 4 Department of Mechanical Engineering, Vocational College, Universitas Gadjah Mada, Jl. Yacaranda Sekip Unit IV, Yogyakarta 55281, Indonesia; 5 Department of Mechanical Engineering, Inha University, 253, Yonghyun-dong, Namgu, Icheon 402-751, Korea

**Keywords:** magnetorheological elastomer, Gr, resistivity properties, rheological properties, force sensing

## Abstract

Considering persistent years, many researchers continuously seek an optimum way to utilize the idea of magnetorheology (MR) materials to be practically used for everyday life, particularly concerning resistivity sensing application. The rheology and resistivity of a graphite (Gr)-based magnetorheological elastomer (Gr-MRE) were experimentally evaluated in the present research. Magnetorheological elastomer (MRE) samples were prepared by adding Gr as a new additive during MRE fabrication. The effect of additional Gr on the rheological and resistivity properties were investigated and compared with those of typical MREs without a Gr additive. Morphological aspects of Gr-MRE were characterized using field emission scanning electron microscopy (FESEM) and energy dispersive X-ray spectroscopy (EDX). Rheological properties under different magnetic fields were evaluated using a parallel-plate rheometer. Subsequently, the resistivity of all samples was measured under different applied forces and magnetic fields. From the resistivity evaluation, two relationship curves resistance (R) under different applied forces (F) and different magnetic fields (B) were established and plotted by using an empirical model. It was observed from the FESEM images that the presence of Gr fractions arrangement contributes to the conductivity of MRE. It was also observed that, with the addition of Gr, rheological properties such as the field-dependent modulus can be improved, particularly at low strain amplitudes. It is also demonstrated that the addition of Gr in MRE can contribute to the likely use of force detection in tactile sensing devices.

## 1. Introduction

The common magnetorheological elastomer (MRE) is generally comprised of micron-size magnetizable particles, matrix, and additives [1]. Carbonyl iron particles (CIP) are most frequently used as magnetic particles owing to their high saturation magnetization of metallic elements as well as high permeability and low remnant magnetization. The particles are dispersed in various types of rubber matrixes such as natural rubber or silicone rubber [2]. As a potential approach to enhancing the properties of MRE, additives such as carbon black [3], multiwall carbon nanotubes [4,5], and nanowires [6] have been used in previous studies. Apart from having magnetic particles embedded in the matrix, a few researchers have utilized other types of particles to further enhance the mechanical and chemical or electrical properties of MREs such as nickel and Gr [7]. 

Previously, Bica et al. [8] used nanoGraphene in the fabrication of a magnetoresistive sensor based on the MRE-hybrid. The nanoGraphene used in the MRE-hybrid were granules between 6 and 8 nm. A viscous electro-conductive solution (SnG) was produced from a mixture of silicone oil and nanoGraphene, which functioned as a main element in the magnetoresistor. The other parts of the magnetoresistor were the resistor body, coupling elements, and magnetoactive elements. The results revealed that the electrical conductivity of the MRE hybrid depended on the intensity of the applied magnetic fields and the amount of compression pressure. The conductivity increased with an increase in the magnetic fields or in the compression pressure. Meanwhile, the electrical conductivity was also investigated using magnetorheological gels (MRGs) by sandwiching a plastic frame containing MRG between two copper (Cu) electrodes to produce a magneto-resistance device. Yu et al. [9] utilized 5, 8, 11, and 14 vol % CIP, and the results revealed that the conductivity of the MRG device increased rapidly with increments in the magnetic fields. For the sample with 14% CIP, the relative change in the conductivity at 1 T was observed to be 300 times higher than that at the off-state condition. It was also observed that the conductive chains became more stable after the MRG sample attained magnetic saturation. Nevertheless, owing to the effective conductive chains formed in the MRG, the resistance decreased rapidly with increment in the CIP volume fractions. Ge et al. [10] also studied the application of conductive MRE in a strain sensor. The MRE was fabricated from a mixture of polydimethylsiloxane (PDMS), carbon nanotube (CNT), and CIP and with/without polyurethane sponge (PUS) reinforcement, wherein typical MR effects were obtained. It was observed that the shear storage modulus increased from 0.49 to 0.64 MPa with PUS reinforcement. The results also revealed that the resistance of the MRE increased with the increase in tensile strain, ranging from 28 to 30.5 kΩ. 

In general, the investigation of electrical resistance includes a resistivity assessment by force analysis. Schûmann et al. [11] analyzed an electro-conductive MRE exhibiting highly complex resistive behavior. The authors used CIPs, carbon black, and silicone as the main ingredients for the MRE fabrication. The results revealed that the resistance increased from 100 to 2300 kΩ and then dropped exponentially to 1200 kΩ at 1 and 3% strain, which was within the linear elasticity region. The authors also analyzed the displacement of the MRE sample, which reduced approximately from 0 to −1.25 mm. Another study by Hreljac et al. [12] demonstrated that the force pressure plate remained the prime definition for detecting force-related events, e.g., in gait detection, which include information regarding heel strike, toe off, and contact times for walking over a range of speeds. According to their study, the limitations of force pressure plates were its static nature and its unsuitability for the constant monitoring of force-related events beyond the laboratory environment. They improved the system by using synchronized force platform recordings of subjects walking at varying speeds. Their results with regard to true average errors (negative for an early prediction) indicated that no systematic errors occurred. Thus, this system could be utilized with 2-D and 3-D kinematic data collection systems. Hanlon et al. [13] demonstrated that the use of force plates was also susceptible to false positives, owing to shifting of weight as they relied on a single calibrated threshold. Apart from the plates, alternative force sensors have been introduced in insole pressure sensors to detect force exerted by the foot [14]. These sensors generally consisted of a 2-D matrix of pressure sensors covering the entire insole. Thus, the need to select numerous sensing points rapidly and accurately, and to simultaneously process them within the time constraints of the application, remains a challenge to be resolved, as commercial sensors possessing high density and high sampling rates are still highly expensive in the market. 

The introduction of Gr powder is likely to generate a new electrical conductivity property while maintaining the existing rheological properties of MRE. The feasibility study on the microstructure and rheological properties of a graphite (Gr)-based magnetorheological elastomer (Gr-MRE) has been conducted by Tian et al. [15]. The authors used 10 g of CIPs, 3 g of silicone oil, and 3 g of silicone rubber with various weight fractions of Gr (0–5 g) for fabricating the MRE. Two types of Gr-MRE were prepared by varying the conditions between isotropic and anisotropic during the curing process; it was observed that the particles were randomly distributed in the isotropic condition, while they were uniformly arrayed as chains in the anisotropic condition. This difference in particle structure contributed toward the increment in the initial storage and loss moduli under the anisotropic condition. In addition, it has been observed that a higher volume fraction of the Gr additive can enhance the MR effect, resulting in higher yield stress under both conditions. In different circumstances, charge transport plays a significant role in the dielectric polarization in the anisotropic composite material containing magnetic particles within the matrix. As such, Moucka et al. [16] employed dielectric spectroscopy to interpret the extent of magnetic filler organization into chain-like clusters formed within the polymer matrix during the curing process. The result revealed that an MRE with 1% CIP content captures more anisotropic spatial distribution as compared to an MRE with 20 wt % CIPs. The weakening of anisotropy in MRE with 20 wt % of CIPs was caused by the growing viscosity of the MRE mixtures before the curing process. Meanwhile, in another study, the author observed that the dielectric relaxations linked to a specific cluster morphology were identified as the manifestation of charge transport by using a variable range hopping (VRH) mechanism along the chain-like structures [17]. However, the study was limited to the investigation of only the rheological properties; the electrical properties such as the conductivity and resistivity of the Gr-MRE in contact with the external input such as the applied force were not considered notwithstanding the significant effect of the Gr on the electric and conductive properties.

A salient benefit of the electrical properties of Gr-MRE could be exploited to resolve the above-mentioned challenging issues. Li et al. [18] introduced a force sensor utilizing Gr-MRE, whose components were 35–65% CIPs, 10–30% Gr particles, and 20–40% silicone rubber. The effect of the magnetic field on the resistivity response of the fabricated samples was tested using an experimental set-up that included mechanical parts, an electrical circuit, and an LED display unit. The results revealed that the prototype force-sensor could be used to detect the external forces at the selected force ranges. However, to the authors’ best knowledge, there has so far been no report on the identification of the relationships among numerous properties, including the rheological, electrical, and magnetic properties, even though such identification would be crucial in enhancing both the accuracy and sensitivity of the Gr-MRE-based force sensor. Consequently, a significant technical contribution consists in the identification of a close relationship among the properties of Gr-MRE samples since it would provide sufficient information for the potential application of Gr-MRE as a force sensor. In order to achieve this goal, a sample of Gr-MRE consisting of CI particles, silicone rubber, and Gr was fabricated, and its microstructure was examined using field emission scanning electron microscopy (FESEM). After analyzing the size of the Gr powder using the particle size analyzer, the effect of material stiffness on the displacement of Gr-MRE was investigated by obtaining data on the resistivity and rheological properties of Gr-MRE. In addition, a new concept different from the method used in [18], for achieving high accuracy, is proposed and validated by establishing the relationship between the resistivity and magnetic properties. The resistivity detection was placed under the contact condition of the Gr-MRE with an external force, whereby the magnetic property was altered by the presence of the magnetic field. The relationship, which can be directly applied to devise the force sensor between the electric resistance and force, was also established in this work. Moreover, in order to highlight the salient properties of the proposed Gr-MRE in the sense of the force sensor, a comparative work between the MRE without the Gr and the Gr-MRE was undertaken to show a higher storage modulus, yield stress, and conductivity and a lower resistance in the proposed sample. 

## 2. Experimental Methods

### 2.1. Materials 

The CIP used in this study was purchased from BASF, Germany, and has an average size of 6 µm. Silicone rubber (SR) at a room temperature of 25 ºC vulcanization (RTV), NS625tds, and silicone oil (SO) DC200 (100cs) were supplied from Nippon Steel Co, Japan, whereas the Gr powder, Gr (code: 8169-00), of an average size of 16 µm and a density of 1.8 gcm^−3^ was obtained from R&M Chemicals, EverGreen Engineering and Resources Co., Malaysia. 

### 2.2. Gr-MRE Fabrication

A series of Gr-MRE samples were synthesized with 12.5 g of CIP, 22.5 g of SR, 20 g of Gr, and 5 g of SO. Initially, the CIP was mixed with SR and SO in a beaker at room temperature of 25 ºC. In the fabrication of MRE samples containing the Gr particles, a sonication process was performed for the Gr particles to break the Van der Waals interaction before being mixed with CIP, SR, and SO. The mixture was stirred for 10 min at 280 rpm to obtain a homogeneous mixture. After that, the Gr was added to the mixture and stirred for another 10 min before a curing agent was added to the mixture to let the sample cure. The curing process followed the procedure provided by the Nippon Steel technical bulletin [19], which explains 2% of the curing agents for the wt % quantity of silicon rubber used. The curing process was done in an off-state condition (no magnetic field) in a mold with a 60 mm diameter and a 1 mm thickness. Finally, the mixture was placed in the mold for curing and left for approximately 1 h. The degassing procedure was then followed to eliminate the presence of air bubbles in the sample. Table 1 presents the components used to fabricate the Gr-MRE. The composition is higher in graphite wt % compared to [15].

### 2.3. Material Characterization

The microstructure of Gr-MRE samples were examined using a field emission scanning electron microscope (FESEM): JSM-7800F PRIME, JEOL, Japan. The samples were coated with platinum and analyzed under 3000× magnification at an accelerating voltage of 4 kV. The size of the Gr powder was examined using a particle size analyzer (PSA): Shimadzu SALD-2300 (WingSALD II: version 3.0.7, Shimadzu Corp. Kyoto, Japan). The Gr powder was dispersed in water to determine the size distribution. Meanwhile, the rheological properties of the Gr-MRE samples were measured using an oscillation rheometer (Physica MCR 302, Anton Paar Company, Graz, Austria). First, a disc with a 20 mm diameter was cut out from the prepared MRE sheet for rheological experiments. A preload of 15 N was applied to the samples before rheological testing, and the sample was then subjected to a 5 N normal force during the experiment in order to avoid wall slip. A magnetic field was generated in the rheometer by a magnetorheological device (MRD 170). The circular sample, ~1 mm, was then placed in the cavity plate. A shear strain in the range 0.001–25% was applied on the sample during the experimental investigation by using a rotary disc parallel plate (pp20) with a diameter of 20 mm and a thickness of 1 mm. 

### 2.4. Rheological and Resistivity Properties

**Figure 1:**
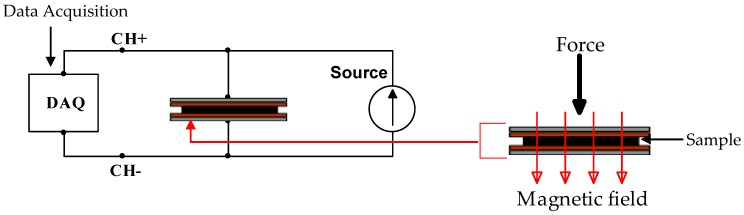
Circuit schematic for resistivity testing (**left**) and fabricated test rig (**right**). DAQ acts as a data logger.

A strain amplitude sweep test and current sweep test were conducted to investigate the dynamic mechanical behavior of the Gr-MRE samples. All the sets of data were collected with different amplitudes of oscillation at various magnetic field strengths. In the sweep strain test, the storage modulus was tested by varying the strain from 0.001 to 25% at different magnetic fields by supplying different current inputs: 0, 1, 2, 3, and 4 A (which are equivalent to 0, 0.18, 0.37, 0.54, and 0.69 T, respectively) at a constant frequency of 1 Hz under a room temperature of 25 ºC. Meanwhile, the sweep current test was performed by supplying continuous current inputs of 0–4 A at a constant strain of 0.01% (to ensure that the strain was in the range of the linear viscoelasticity) and a constant frequency of 1 Hz at room temperature. The MR effect formula is expressed in Equation (1) as follows:

(1) $$\text{MR effect}=\frac{G_{max}-G_{0}}{G_{0}}\times 100\%$$

where G_max_ is the maximum modulus, and G_0_ is the initial modulus [20]. The schematic of the experimental setup to study the resistance of the Gr-MRE is shown in Figure 1.

Before the resistivity test was carried out, the hardness of the samples was determined first by using elastomer durometer shore A (HBA 100-0: Sauter GmbH, Balingen, German). The hardness test measures the depth of the presser foot indented in the material by the force exerted onto the sample. The resistivity of the Gr-MRE was observed using a straightforward test rig that was set up to determine the resistance, from the exerted forces. Nonmagnetic slotted weights (see Figure 1) in the range of 100–1000 g was used as a force resource in this experiment. The data acquisition (DAQ) used was obtained from LabVIEW (6211, National Instruments, Austin, TX, USA). After the connection was set up, the off-state test (without magnetic field) was conducted by placing a 100 g weight on the Gr-MRE, and the force given was distributed equally on the entire surface of the tested sample. The test was then repeated with higher weights at intervals of 100 g. The on-state testing (with magnetic field) was carried out in a similar condition, but the Gr-MRE was sandwiched between permanent magnets. These magnets were used to induce magnetic fields in the Gr-MRE in order to investigate the change in the mechanical properties of the material. 

## 3. Results and Discussion

### 3.1. FESEM Characterization

**Figure 2:**
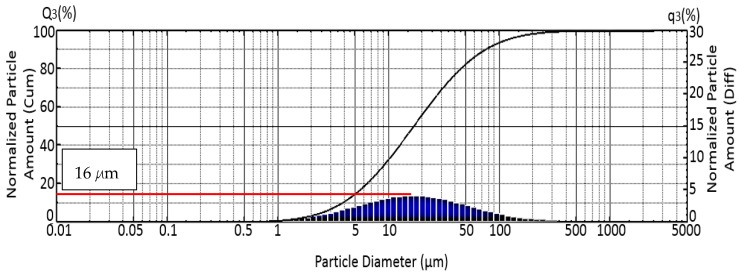
Particle size analyzer results for Gr powder. The red line shows the average size of graphite particles.

**Figure 3:**
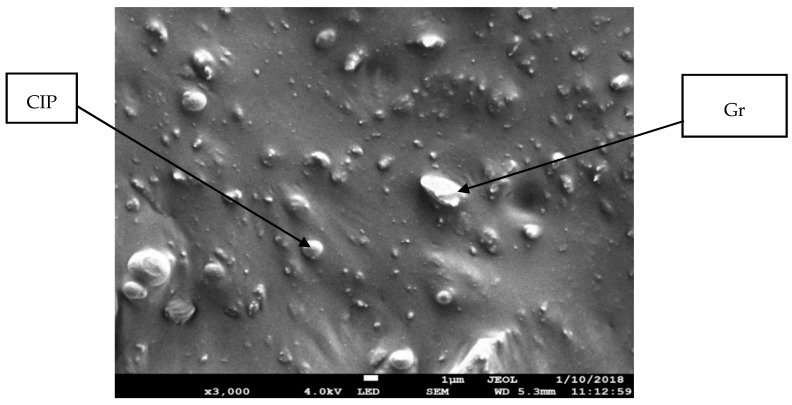
Microstructure of the Gr-MRE (Gr 33%). Gr particles are irregular, while CIPs have a spherical shape.

Figure 2 illustrates the particle size analysis of the Gr powder. The result reveals a normal distribution with an average size of powder particles of 16 µm. In previous studies, Li et al. [18] used Gr particles with 12 µm, and Tian et al. [15] used 20 µm Gr particles where Li et al. [18] reported that the size of the Gr particles affects the resistivity of the MRE. Therefore, in this study, the size of Gr used was 16 µm, which is in the range of the particle size used by previous researchers. Figure 3 displays the microstructure of the Gr-MRE sample. The figure shows that the CIP particles were randomly distributed in the rubber matrix, whereas the Gr particles were distributed randomly between the CIP particles albeit in different orientations as a result of its natural nonlinear shapes and sizes. Figure 3 also shows that the Gr-MRE mix appeared as bright points or lines in the micrographs as higher quantities of Gr were added to the MREs [15]. Although Gr constitutes 33% of the Gr-MRE, only a few Gr particles were noticeable because most of the Gr particles were embedded in different orientations in the matrix. The surface of the Gr-MRE was also observed to be rough because of the cutting process.

**Figure 4:**
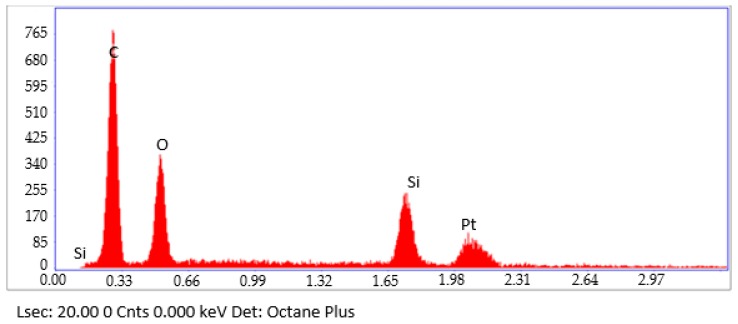
Carbon has the highest peak among all compositions in the Gr-MRE during EDX observations.

Figure 4 shows the EDX results obtained from the FESEM images. Based on the FESEM micrograph, a few points were selected to determine the composition of the Gr-MRE. Carbon exhibits the highest proportion, followed by platinum, silicon, and oxygen (34.35, 29.71, 20.64, and 15.31%, respectively). The weights and atomic percentages are tabulated in Table 2. Sengupta et al. [21] mentioned that Gr particles are made of two-dimensional lattice bonds, the most stable form of carbon under standard conditions; this explained the status of carbon as the largest constituent in the EDX results. Platinum was used in the coating process conducted before the FESEM observation of the Gr-MRE sample; this was to prevent the charging of the sample surface with electrons, which causes glare images. Since SR was used as the matrix for Gr-MRE fabrication, silicon could also be distinguished.

#### 3.1.1. The Effect of Gr on Storage Modulus

**Figure 5:**
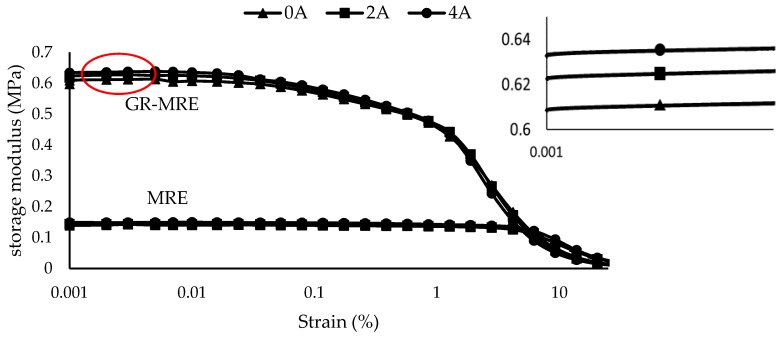
Storage modulus of MRE and Gr-MRE at different strain amplitude at different magnetic fields of 0, 2, and 4 A.

Figure 5 shows the storage modulus of the MRE filled with Gr, at various strain amplitudes and current sweeps. Under an oscillating force, the strain amplitude depends on both the elastic and viscous behaviors of the materials [2]. The elastic modulus of SR is related to the storage modulus, which measures the recoverable energy in the deformed sample. It can be observed that, as the strain amplitude increases, the storage modulus decreases. The non-linear behavior shown in this result is generally known as the Payne effect, which is a feature of a behavior of rubber containing fillers, and this could be explained by the existence of a filler structure in the rubber matrix above the percolation threshold [22]. Commonly the storage modulus of a filled composite is influenced by the effective interfacial interaction between the filler and the matrix [5]. The agglomeration in fact will lower the storage modulus [23]. The initial stiffness achieved by the samples follow the theoretical model of particle-filled rubber, which has been introduced by Guth [24]; G* = G_i_(1 + 2.5Φ + 14.1Φ^2^), where G* is the filled rubber modulus, G_i_ is the initial modulus, and Φ is the volume fractions of the filler. With the addition of filler in the rubber, the storage modulus increased accordingly [25]. In general, a stronger interaction results in a higher storage modulus of the composite. In all cases, the initial modulus of the MRE filled with Gr was higher at all the magnetic fields in comparison with the MRE sample without Gr. However, the linear region of the Gr-MRE samples are lower than that of the MRE sample, which indicates that Gr-MRE has a higher storage modulus at low strain amplitude [15]. An analysis of the slopes and curves reveals that, as the Gr fraction in a material increases, the growth of the slopes decreases parallel with the increment of the magnetic field—from 0 to 0.245 T. This is due to the contributions of the Gr powders to the stiffness of the samples. As shown in Figure 5, the storage modulus exhibits an approximately linear relationship with strain before deformation, and this result is consistent with the finding by Tian et al. [15], who found the same pattern of a higher initial modulus in the graphite-based MRE, as compared to the conventional MRE. However, for the conventional MRE, the storage modulus exhibits an almost identical trend at all magnetic field intensities. 

#### 3.1.2. The Effect of Gr-MRE on Loss Factor

**Figure 6:**
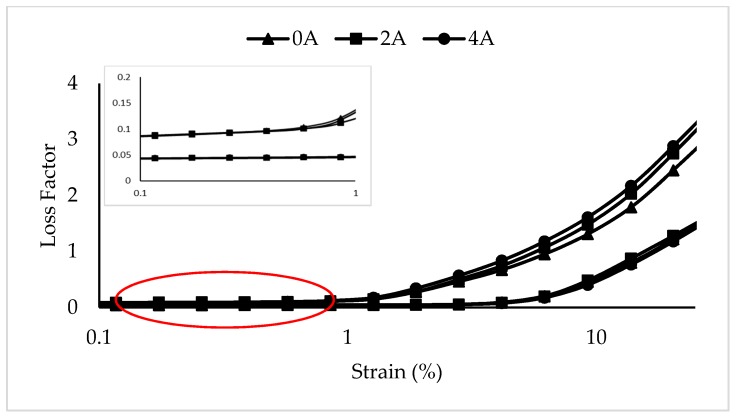
Loss factors of MRE and Gr-MRE at different strain amplitudes at magnetic fields of 0, 2, and 4 A.

The variation in loss factor or tan ∂ of the MRE and Gr-MRE as a function of strain, obtained through the sweep strain test, is shown in Figure 6. Small groups and chains and the movement of the molecules within the polymer structure affect the loss factor. Therefore, the higher the loss factor, the higher the degree of molecular mobility. The phenomena wherein the Gr-MRE samples exhibit a higher loss factor compared to the MRE without Gr, starting from a lower strain amplitude at 0.1% all the way to 25%; this indicates a large degree of molecular mobility. The Gr incorporated as filler in the matrix functions as barriers to the mobility of the matrix chains throughout the strain amplitude. As the weight fraction of Gr increases, the movement of the particles in the matrix is limited, and this leads to a sample with a higher loss factor, with strain starting at 1% and increasing to 25%. Tian et al. [15] observed a similar overall trend, which demonstrates that the MRE without Gr has a lower loss factor compared to the MRE with Gr. The higher loss factor of the Gr-MRE compared to the MRE is likely to be because of the interconnected network between Gr and CIP particles in the matrix.

#### 3.1.3. The Effect of Gr Presence in MRE on Frequency

**Figure 7:**
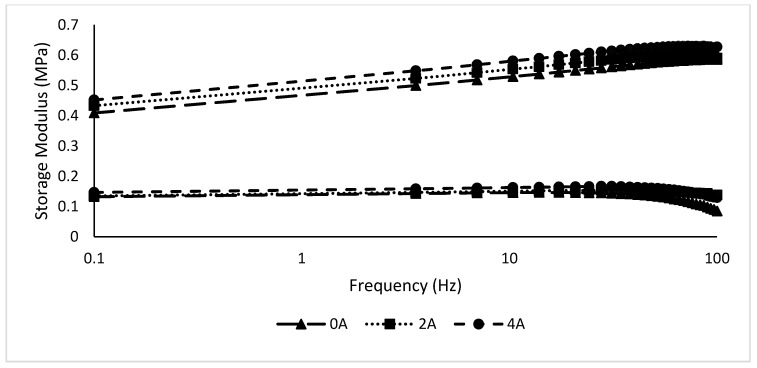
Storage modulus of MRE and Gr-MRE at different frequency amplitudes at magnetic fields of 0, 2, and 4 A.

**Figure 8:**
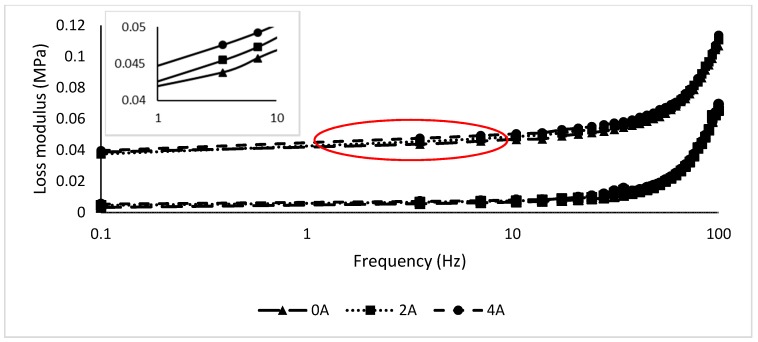
Loss modulus of MRE and Gr-MRE at different frequency amplitudes at magnetic fields of 0, 2, and 4 A.

In this test, the strain is set at 0.01% based on the linear viscoelastic region during the strain sweep test. The frequency varied from 0 to 100 Hz at 0, 2, and 4 A. Figure 7 and Figure 8 show the storage and loss moduli of the Gr-MRE and conventional MRE samples. 

As shown in Figure 7, the storage modulus of all Gr-MRE exhibited an increasing trend with the growth of frequency and decreased when approaching 60 Hz. In comparison to the conventional MRE samples, the Gr-MRE shows higher initial and maximum storage moduli. This trend can be related to the existence of Gr in the MRE samples. This is also believed to be due to the two-dimensional structures of Gr and the good mixing of GR in MRE. Meanwhile, the loss moduli of all samples in Figure 8 along with the inset show an insignificant effect toward the frequency regardless of the addition of Gr in the MRE and appear to have elevated logarithmically starting from 10 to 100 Hz. This resulted depicts that, at high frequency, more energy dissipated from the sample. 

#### 3.1.4. The Effect of Gr Presence in MRE on the MR Effect

**Figure 9:**
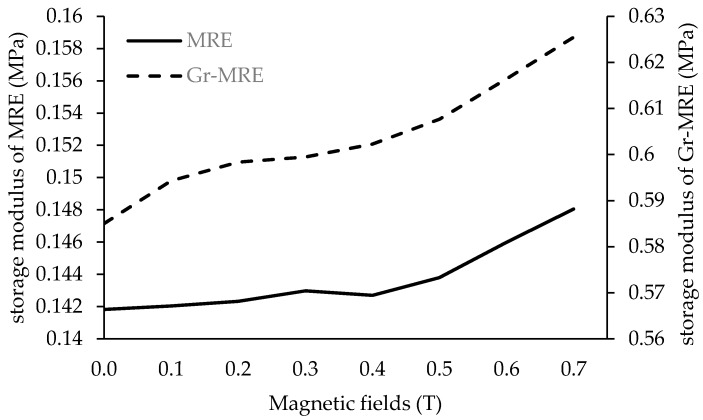
The MR effect of MRE and Gr-MRE samples at magnetic fields of 0–0.7 T.

Figure 9 shows the storage modulus versus the current sweep of the MRE with and without Gr. ΔG and the MR effect are presented in Table 3. By adding Gr in the MRE sample, the MR effect was increased by 3% compared to the MRE without Gr. The incorporation of Gr in the composite enhanced the MR effect. In the MRE, Gr functioned as the conductive agent and contributed to the increment in storage modulus. The storage modulus increased because Gr particles contributed to the initial stiffness of the Gr-MRE [15]. With the increase in the initial stiffness of the sample, the stiffness change that influences the MR effect will be different from that of the conventional MREs.

**Figure 10:**
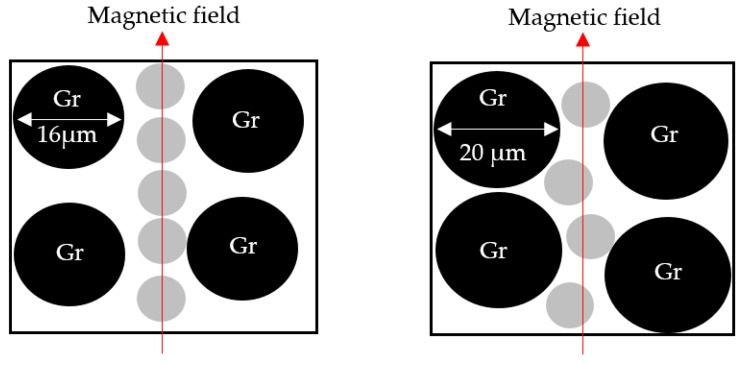
The difference between graphite content used by Tian et al, 20 µm (**right**), and current research, 16 µm (**left**). The increase in particle size limits particle mobility.

Further factors that possibly influence the MR effect are the matrix, the magnetic particle, the graphite properties, and the curing methods. Therefore, in order to identify the effect of each component, a set of experimental works was carried out. Similar to previous work [15], the results showed that the MR effect had slightly different values (2.6%). Figure 10 shows the concentration of Gr used in the MRE fabrication. The MR effect of the current/previous work might be lower because the increased graphite content contributed to a higher initial storage modulus [15], and this leads to a higher stiffness. Another possibility is that the RTV silicone rubber matrix provides more room for CIP particles to vibrate and thus introduces a sample that is more elastic [26]. This would increase the MR effect and provide suitable curing conditions that randomly distributed the composite particles throughout the matrix. Although the detail of the previous curing condition has not been comprehensively described, it is well known that curing conditions have some differences, especially in terms of the particle alignment caused by the magnetic field applied during the curing process. Such composite (anisotropic) exhibits different physicochemical behavior such as higher MR efficiency [27]. Nevertheless, the investigation of each component is beyond the scope of this paper but could be investigated further in the future.

### 3.2. Resistivity Properties

Variation was observed in the hardness of the Gr-MRE compared to the conventional MRE. The Gr-MRE shows a higher hardness value of 61, which is 20% stiffer as compared to the conventional MRE, which has a hardness value of 51. 

**Figure 11:**
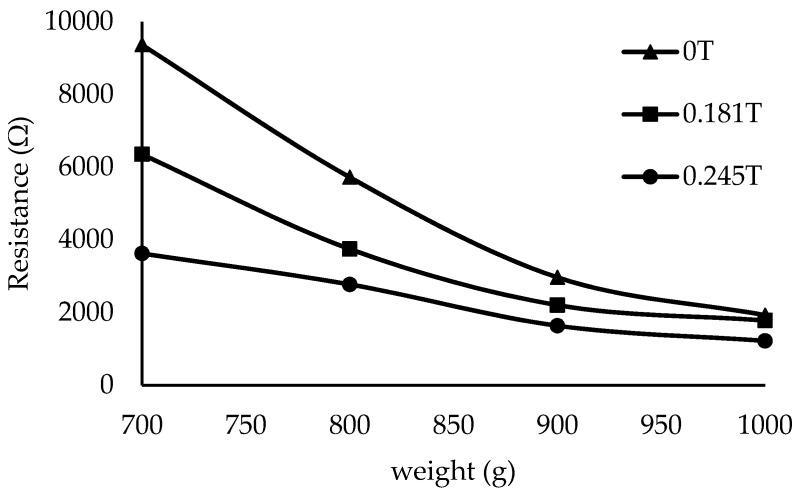
Resistance reduction of MRE samples with graphite content at magnetic fields of 0, 0.181, and 0.245 T.

Figure 11 shows the magnitude of the resistance (R) versus the applied weight for various magnetic fields. The experiment was conducted by placing weights of 100–1000 g at increments of 100 g, with the magnetic field varying from 0 to 0.245 T, but the reading was only detectable at 700–1000 g. The magnitudes of R were recorded. R decreased exponentially as the weight changed from 700 to 1000 g. Under the magnetic field, under all applied forces and conditions, the magnitude of R showed a decreasing trend. R displayed a decreasing trend as the weight varied from 700 to 1000 g: 9367–1929 Ω, 6353–1782 Ω, 5393–1528 Ω, and 3623–1221 Ω at 0, 0.181, 0.214, and 0.245 T, respectively. The decreasing trend of R with increasing weight explains the field-induced particle motion. When the magnetic field increases, it would increase the particle magnetization and the attractive forces between the particles. These particles tend to form a chain structure resulting in the increment in conductivity and reduction in R. The results are consistent with the observations of Li et al. [18]; in this work, the authors state that the rate of decrease of R. is higher than 60% when the applied force is increased. At 0, 0.181, 0.214, and 0.245 A, the reductions in R were observed to be approximately 79, 72, 71, and 66%, respectively. This phenomenon demonstrates that, when the magnetic field intensity increases, R reduces. This phenomenon might be because under various magnetic fields, the CIP content was affected by particle mobility, thereby affecting the movement of Gr particles. Moreover, MRE also exhibits piezoresistivity. When an MRE sample is compressed, its conductivity increases because of two factors: the increment of the conductive area induced by the deformation of the MRE and the reduction in the thickness of the polymer membrane between two adjacent particles [28]. As widely known, the R value has a proportional relationship with the length of a cable or, in this work, the thickness of the sample and an inversely proportional relationship with the conductive area of the sample. The same phenomena and conclusion have also been shown by Tian et al. [28].

**Figure 12:**
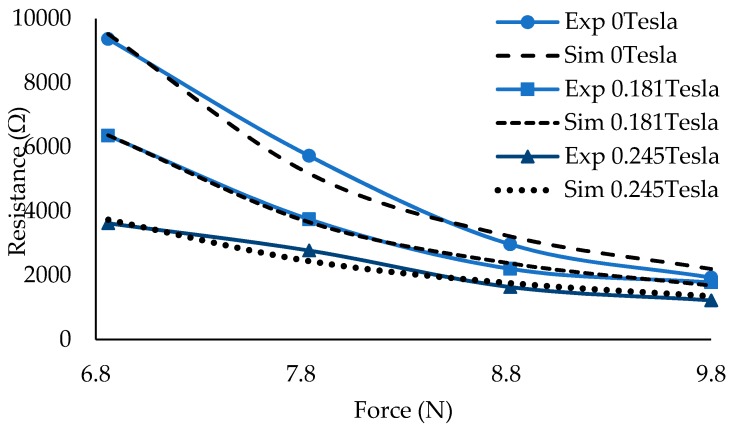
Resistance reduction showed by the simulation and experimental data at different force applied.

The relationship between the resistance value and the applied force can be analyzed and characterized using an empirical model. The relationship between the applied forces or mass and R in the proposed work and other works that deal with the smart materials affected by resistance and magnetic fields, such as that by Li et al. [18], is similar to the decay phenomena described by the Arrhenius equation [29]. The decrease in R as a function of the weight or force (F) can be described using Equation (2), where $A_{0}$ is a material constant, and Q is another constant that replaces the activation energy in the Arrhenius equation. Figure 12 shows an example wherein the model is fitted to the experimental results for two magnitudes of the magnetic field, the off-state condition (0 T), and 0.181 T. The figure shows a reasonable agreement between the simulation and experimental results. More comprehensive observations and accuracies are described in Table 4. The $A_{0}$ and Q values are decreased with the increase in magnetic fields because, in general, the resistance values are also decreased with the increase in the applied magnetic fields. Hence, the root mean square errors (RMSEs) also tend to be lower at higher magnetic field intensities. Meanwhile, the correlation between the resistance and force can be observed when R^2^ changes. In general, the correlations are acceptable, with values being over 0.9500. Based on Equation (2) and previous discussions, $A_{0}$ and Q values can be assumed to have dependency on the initial stiffness of the material. A higher magnetic field means that the material will become stiffer. Consequently, the thickness difference before and after more weight of the material is added at higher magnetic fields is less than that at a lower magnetic field. In other words, stiffer material means that $A_{0}$ becomes higher, as shown in Table 4. If the model is applicable to a wider range of the applied force, the resistance value is expected to be nearly constant. One of the reasons can be that the maximum allowable compression has been reached, but this phenomena need to be further validated.

(2) $$R\left(F\right)=A_{0}\;exp\left(\frac{Q}{F}\right).$$

**Figure 13:**
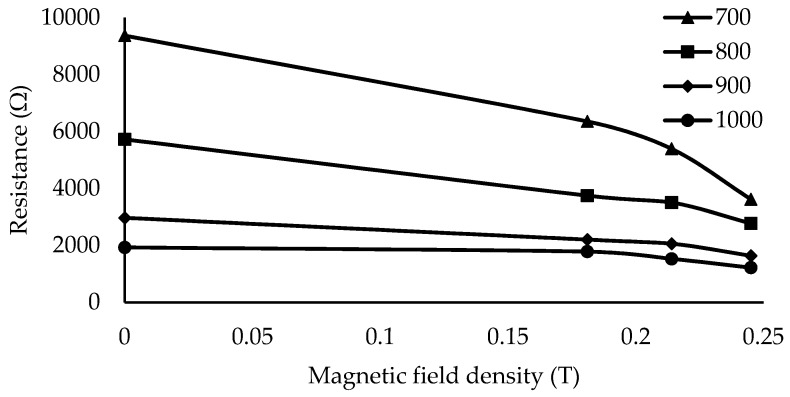
Resistance reduction in different magnetic fields at applied forces of 700, 800, 900, and 1000 g.

Meanwhile, the effect of the magnetic field on the resistance value is interesting to discuss. Figure 13 shows the reduction in resistance for specific applied weights, under magnetic fields from 0 to 0.245 T. It is evident that R decreased as the magnetic field strength increased. The observed behavior and pattern is similar to that observed in the study by Li et al. [18]. It is apparent that the Gr particles introduced conductivity in the MRE; thus, the output was detected as the change in resistance. While the increase in the Gr weight fraction increases the conductivity, it reduces the resistivity when the material is subjected to an external applied force.

## 4. Conclusions

In this study, MREs filled with Gr were fabricated, and material characterization was undertaken by investigating the relationships between the rheological property and magnetic field intensity, the resistivity and magnetic field, the resistance and force, etc. From the overall experimental data and results, the following observations were made:

(1) The storage modulus of Gr-MRE was increased by the effective interfacial interaction between the filler and matrix, where a stronger interaction resulted in a higher storage modulus of the Gr-MRE in comparison with the conventional MRE [30]. 

(2) The Gr-MRE samples exhibited a higher loss factor compared to the MRE without Gr at strain amplitudes from 0.1 to 25%; this indicates a high degree of molecular mobility. The incorporated Gr functioned as a filler in the matrix for all the strain amplitudes. 

(3) The MR effect of the Gr-MRE became 60% higher than that of the conventional MRE and 176% higher than that of the previous Gr-MRE study. In the MRE, Gr functioned as the conductive agent and contributed to the increment of the yield stress corresponding to the storage modulus. 

(4) The increment in the magnetic field increased the particle magnetization and the attractive forces between the particles. These particles tended to form a chain structure, resulting in an increment in the conductivity and a reduction in the resistance. The Gr particles provided conductivity to the MRE; therefore, the output was detected as the change in resistance, which had a close relationship with the external force generated from the contact with the Gr-MRE.

The results presented in this work are more or less self-explanatory, justifying that the MRE filled with Gr can detect up to 10 N of applied force at a certain magnetic field intensity. This indicates that the controllable conductivity of Gr-MRE can be a potential candidate for applications in force sensors, particularly for the measurement of contact force. The correlation models characterizing the resistivity properties as a function of magnetic fields and applied forces are to be developed to demonstrate the practical feasibility of the Gr-MRE-based force sensor. This will be undertaken as a second phase of this work in the near future. 

## Figures and Tables

**Table 1:** Components of the graphite (Gr)-based magnetorheological elastomer (Gr-MRE) sample.

Compound Elements	Weights (g)	wt %
Carbonyl iron (6 µm)	12.5	21
Silicon rubber	22.5	37
Gr (16 µm)	20	33
Silicone oil	5	9

**Table 2:** Elements in the Gr-MRE.

Element	Weight (%)	Atomic (%)
C	34.35	60.80
O	15.31	20.34
Si	20.64	15.62
Pt	29.71	3.24

**Table 3:** The MR effect on the storage modulus of the MRE samples.

Samples	G′_0_	G′_max_	ΔG′	MR Effect
**Conventional MRE (CIP: 20 wt %)**	0.1418	0.1482	0.0064	4.5%
**Gr-MRE (Tian et al.** [15]) **(CIP: 47.61 wt %)**				2.6%
**Gr-MRE (CIP: 20 wt %)**	0.5850	0.6272	0.0422	7.2%

**Table 4:** Values of parameters at various magnetic fields based on Equation (2).

B	$A_{0}$ (Ω)	Q(N)	RMSE (Ω)	R^2^
0	72.16	33.49	340.52	0.986
0.181	75.52	30.42	109.24	0.996
0.214	90.29	28.14	144.56	0.991
0.245	126.14	23.24	195.17	0.957
0.312	177.97	18.24	44.54	0.993

## Abbreviations

Gr-MRE	Gr-based magnetorheological elastomer
MR	Magnetorheological
MRE	Magnetorheological elastomer
MRF	Magnetorheological fluid
Oe	Oersted
Wt%	Weight percent
RTV	Room temperature vulcanized
CIPs	Carbonyl iron particles
SR	Silicone rubber
SO	Silicone oil
DMM	Digital multimeter
DAQ	Data acquisition

## References

[1] Gong X.L., Zhang X.Z., Zhang P.Q. (2005). Fabrication and characterization of isotropic magnetorheological elastomers. Polym. Test..

[2] Sutrisno J., Purwanto A., Mazlan S.A. (2015). Recent progress on magnetorheological solids: Materials, fabrication, testing, and applications. Adv. Eng. Mater..

[3] Abdullateef A.A., Thomas S.P., Al-Harthi M.A., De S.K., Bandyopadhyay S., Basfar A.A., Atieh M.A. (2012). Natural Rubber Nanocomposites with Functionalized Carbon Nanotubes: Mechanical, Dynamic Mechanical, and Morphology Studies. J. Appl. Polym. Sci..

[4] Li R., Sun L.Z. (2011). Dynamic mechanical behavior of magnetorheological nanocomposites filled with carbon nanotubes. Appl. Phys. Lett..

[5] Aziz S.A.A., Mazlan S.A., Ismail N.I.N., Ubaidillah U., Choi S.B., Khairi M.H.A., Yunus N.A. (2016). Effects of multiwall carbon nanotubes on viscoelastic properties of magnetorheological elastomers. Smart Mater. Struct..

[6] Antonel P.S., Oliveira C.L.P., Jorge G.A., Perez O.E., Leyva A.G., Negri R.M. (2015). Synthesis and characterization of CoFe_2_O_4_ magnetic nanotubes, nanorods and nanowires. Formation of magnetic structured elastomers by magnetic field-induced alignment of CoFe_2_O_4_ nanorods. J. Nanopart. Res..

[7] Zou H., Zhang L., Tian M., Wu S., Zhao S. (2009). Study on the structure and properties of conductive silicone rubber filled with nickel-coated graphite. J. Appl. Polym. Sci..

[8] Bica I., Anitas E.M., Bunoiu M., Vatzulik B., Juganaru I. (2014). Hybrid magnetorheological elastomer: Influence of magnetic field and compression pressure on its electrical conductivity. J. Ind. Eng. Chem..

[9] Yu M., Yang P., Fu J., Liu S., Choi S.B. (2016). A theoretical model for the field-dependent conductivity of magneto-rheological gels and experimental verification. Sens. Actuators A Phys..

[10] Ge L., Gong X., Wang Y., Xuan S. (2016). The conductive three dimensional topological structure enhanced magnetorheological elastomer towards a strain sensor. Compos. Sci. Technol..

[11] Schümann M., Morich J., Kaufhold T., Böhm V., Zimmermann K., Odenbach S. (2018). A mechanical characterisation on multiple timescales of electroconductive magnetorheological elastomers. J. Magn. Magn. Mater..

[12] Hreljac A., Marshall R.N. (2000). Algorithms to determine event timing during normal walking using kinematic data. J. Biomech..

[13] Hanlon M., Anderson R. (2009). Real-time gait event detection using wearable sensors. Gait Posture.

[14] Peng Z., Cao C., Huang J., Pan W. (2013). Human moving pattern recognition toward channel number reduction based on multipressure sensor network. Int. J. Distrib. Sens. Netw..

[15] Tian T.F., Li W.H., Alici G., Du H., Deng Y.M. (2011). Microstructure and magnetorheology of graphite-based MR elastomers. Rheol. Acta.

[16] Moucka R., Sedlacik M., Cvek M., Moucka R., Sedlacik M., Cvek M. (2018). Dielectric properties of magnetorheological elastomers with different microstructure. Appl. Phys. Lett..

[17] Moučka R., Sedlačík M., Kutálková E. (2018). Magnetorheological Elastomers: Electric Properties versus Microstructure. AIP Conf. Proc..

[18] Li W., Kostidis K., Zhang X., Zhou Y. Development of a Force Sensor Working with MR Elastomers. Proceedings of the 2009 IEEE/ASME International Conference on Advanced Intelligent Mechatronics.

[19] Nippon Steel Nippon Steel Technical Bulletin ns625tds. http://www.yureka.com.my/file/Technical%20Specs%20PDF/ns%20625%20tds.pdf.

[20] Li W.H., Zhang X.Z., Du H. (2013). Magnetorheological elastomers and their applications. Adv. Elastomers.

[21] Sengupta R., Bhattacharya M., Bandyopadhyay S., Bhowmick A.K. (2011). A review on the mechanical and electrical properties of graphite and modified graphite reinforced polymer composites. Prog. Polym. Sci..

[22] Ponnammaa S.T.D., Sadasivunib K.K., Grohensc Y., Guod Q. (2014). Carbon Nanotubes based Elastomer Composites—An Approach towards Multifunctional Materials. J. Mater. Chem. C.

[23] Inam F., Wong D.W.Y., Kuwata M., Peijs T. (2010). Multiscale Hybrid Micro-Nanocomposites Based on Carbon Nanotubes and Carbon Fibers. J. Nanomater..

[24] Guth E. (1945). Theory of filler reinforcement. J. Appl. Phys..

[25] Choi H.J., Mazlan S.A., Imaduddin F. (2016). Fabrication and viscoelastic characteristics of waste tire rubber based magnetorheological elastomer. Smart Mater. Struct..

[26] Boczkowska A., Awietj S. (2012). Microstructure and Properties of Magnetorheological Elastomers. Adv. Elastomers Technol. Prop. Appl..

[27] Jung H.S., Kwon S.H., Choi H.J., Jung J.H., Kim G. (2015). Magnetic Carbonyl Iron / Natural Rubber Composite Elastomer and Its Magnetorheology. Compos. Struct..

[28] Tian T.F., Li W.H., Deng Y.M. (2011). Sensing capabilities of graphite based MR elastomers. Smart Mater. Struct..

[29] Bahiuddin I., Mazlan S.A., Shapiai I., Imaduddin F., Choi S.B. (2018). Constitutive models of magnetorheological fluids having temperature-dependent prediction parameter. Smart Mater. Struct..

[30] Yunus N.A., Mazlan S.A., Choi S.B., Imaduddin F., Aziz S.A.A., Khairi M.H.A. (2016). Rheological properties of isotropic magnetorheological elastomers featuring an epoxidized natural rubber. Smart Mater. Struct..

